# SIRT1 Interacts with and Deacetylates ATP6V1B2 in Mature Adipocytes

**DOI:** 10.1371/journal.pone.0133448

**Published:** 2015-07-15

**Authors:** Sun-Yee Kim, Qiongyi Zhang, Reinhard Brunmeir, Weiping Han, Feng Xu

**Affiliations:** 1 Singapore Institute for Clinical Sciences, Agency for Science, Technology and Research (A*STAR), Singapore, Singapore; 2 Laboratory of Metabolic Medicine, Singapore Bioimaging Consortium, A*STAR, Singapore, Singapore; 3 Department of Biochemistry, Yong Loo Lin School of Medicine, National University of Singapore, Singapore, Singapore; Boston University School of Medicine, UNITED STATES

## Abstract

SIRT1 plays a key role in maintaining metabolic homeostasis in mammals by directly modulating the activities of various transcription factors and metabolic enzymes through lysine deacetylation. White adipose tissue plays a key role in lipid storage and metabolism. To identify novel molecular targets of SIRT1 in fat cells, we used a non-biased proteomic approach. We identified a number of proteins whose acetylation status was significantly affected by SIRT1 modulator treatment in 3T3-L1 adipocytes. Among them, ATP6V1B2, a subunit of the vacuolar (H^+^)-ATPase, was further shown to be associated with SIRT1 by co-immunoprecipitation assay. Moreover, SIRT1 deacetylates ATP6V1B2 *in vitro* and *in vivo*. Taken together, our study demonstrates that ATP6V1B2 is a molecular target of SIRT1 in fat cells and the role of SIRT1 and ATP6V1B2 acetylation in the vacuolar (H^+^)-ATPase function warrants further investigation.

## Introduction

SIRT1 is a representative member of the mammalian sirtuin family that deacetylates a wide variety of protein substrates in an NAD^+^-dependent manner. It was originally identified as a histone deacetylase [1, 2] but was later shown to target many important regulators such as p53, PPAR-γ, FOXO proteins [3] and PGC1α [4]. By deacetylating the tumor suppressor protein p53 and the FOXO family of transcription factors, SIRT1 modulates the activity of these regulators and controls cell proliferation and stress resistance [5, 6]. In addition to these functions, SIRT1 has a major role in the mammalian calorie restriction response [7, 8]. Moreover, SIRT1 participates in the regulation of multiple metabolic processes such as glucose homeostasis [4], insulin secretion [9], insulin sensitivity [10] and fatty acid β-oxidation (FAO) [11–13] by modulating the expression or activity of critical transcriptional regulators of these processes, including PGC1α [4, 11, 13], UCP2 [9], PTP1B [10], PPAR-γ [14] and PPAR-α [12], in several organs.

Lysine acetylation is a conserved protein post-translational modification (PTM). An acetyl group can be added to the end of the side chain of a lysine residue via the action of protein acetyltransferases and removed via the action of protein deacetylases. Lysine acetylation was originally identified in histones 50 years ago [15], but its role in eukaryotic gene activation was discovered decades later [16]. Moreover, the existence and function of lysine acetylation in non-histone proteins are increasingly attracting attention. Recently, a number of acetylome studies significantly expanded our knowledge regarding this PTM by identifying thousands of novel acetylation sites and hundreds of novel acetylated proteins [17–20]. Importantly, Zhao et al. reported that virtually every enzyme involved in glycolysis, gluconeogenesis, fatty acid metabolism and glycogen metabolism is acetylated in human liver tissue [18], indicating that lysine acetylation may have a much broader role in metabolic regulation than previously expected. In contrast to the predominant permissive role of histone acetylation, lysine acetylation of non-histone proteins can be either permissive or non-permissive. For example, although acetylation activates p53 sequence-specific DNA binding, PGC1α is repressed when it is acetylated by the acetyltransferase Gcn5 [21] and activated by SIRT1 deacetylation [4]. Moreover, the activities of various metabolic enzymes are also modulated by the lysine acetylation status. For example, the enzymes enoyl-CoA hydratase/3-hydroxyacyl-CoA (EHHADH) and malate dehydrogenase (MDH) exhibited greater activity when acetylated, while the activity of argininosuccinate lyase (ASL) was inhibited by lysine acetylation [18].

SIRT1 exerts its regulatory function mainly through two mechanisms. First, it regulates gene expression by modulating the histone acetylation state and thus controls the levels of various downstream targets [14]. Second, it directly modulates the activities of various enzymes and transcription factors by lysine deacetylation [4, 5, 11]. It is evident that many nuclear transcription factors are substrates for SIRT1 deacetylation, and their transcriptional activity is therefore under the control of SIRT1. Furthermore, a few studies have shown that SIRT1 could be exported to the cytoplasm to deacetylate metabolic enzymes and modulate their activities [9, 22, 23].

In mammals, white adipose tissue (WAT) is the main site for lipid storage, and it plays a key role in lipid metabolism and energy production. SIRT1 is also expressed in the WAT of mice [14]. We thus chose white adipocytes to further explore the molecular targets of SIRT1 aiming to identify novel roles of this enzyme in fat cells. Toward this end, we adopted a proteomic approach to identify novel substrates of SIRT1 deacetylation in *in vitro* differentiated 3T3-L1 adipocytes. Unexpectedly, ATP6V1B2, a subunit of the vacuolar (H^+^)-ATPase, was identified as a potential SIRT1 target. We further demonstrated that SIRT1 directly interacts with and deacetylates ATP6V1B2 *in vitro*, thus adding ATP6V1B2 to the list of SIRT1 targets.

## Materials and Methods

### Cell Culture, Differentiation and Treatment

Human embryonic kidney (HEK) 293 cells and mouse 3T3-L1 preadipocytes were purchased from the American Type Culture Collection (ATCC) and cultured in DMEM containing 10% bovine calf serum (BCS). *In vitro* adipogenic differentiation of 3T3-L1 cells was carried out essentially as described [24]. SIRT1 inhibitor (EX527), SIRT1 activator (SRTCX1002), and placebo (SRTCZ1001) were provided by GlaxoSmithKline (GSK). To validate the SIRT1 modulators, HEK293 cells were treated with 10 μM of these small molecules as follows: (I) DMSO for 48 hours; (II) EX527 for 48 hours; (III) EX527 for 24 hours then switched to SRTCX1002 for 24 hours; or (IV) EX527 for 24 hours then switched to SRTCZ1001 for 24 hours. The cells were then harvested for western blot analyses. To examine the effects of SIRT1 modulator treatment on mature adipocytes, 3T3-L1 cells at Day 6 of adipogenesis were treated as described above for HEK293 cells; they were then harvested for further analyses.

### Two-Dimensional Gel Electrophoresis (2-DE)

Whole-cell lysates (WCLs) from SIRT1 modulator-treated 3T3-L1 mature adipocytes were prepared and subjected to 2-DE separation according to the carrier ampholine method of isoelectric focusing (IEF) [25, 26] by Kendrick Labs, Inc. (Madison, WI). Isoelectric focusing was carried out in a glass tube with an inner diameter of 3.3 mm using 4 L of 2.0% Servalyt at pH 3.5–10 (Serva, Heidelberg, Germany) and 2 mM lysine for 20,000 volt-hours. One microgram of an IEF internal standard, tropomyosin, was added to each sample. This protein migrates as a doublet with a lower polypeptide spot with a molecular weight (MW) of 33,000 and a pI of 5.2. The enclosed tube gel pH gradient plot for this set of ampholines was determined with a surface pH electrode.

After equilibration for 10 minutes in Buffer 'O' (10% glycerol, 50 mM dithiothreitol, 2.3% SDS and 0.0625 M Tris, pH 6.8), each tube gel was sealed to the top of a stacking gel that overlaid a 10% acrylamide slab gel (1.00-mm thickness). SDS slab gel electrophoresis was carried out for approximately 5 hours at 25 mA/gel. After electrophoresis was complete, duplicate gels for blotting were placed in transfer buffer (10 mM CAPS, pH 11.0, 10% MeOH) and transblotted onto PVDF membranes overnight at 225 mA with approximately 100 volts/two gels. The following proteins (Sigma, St. Louis, MO) were used as MW standards: myosin (220,000), phosphorylase A (94,000), catalase (60,000), actin (43,000), carbonic anhydrase (29,000) and lysozyme (14,000). These standards appear as bands at the basic edge of the Coomassie Brilliant Blue R-250-stained membrane. These stained blots were then desktop-scanned and probed with a pan-acetyl-lysine antibody (Cat # 9441, Cell Signaling).

### Image Visualization and Protein Identification

Duplicate acetyl-lysine western blot images were obtained for each sample and scanned with a laser densitometer (Model PDSI, Molecular Dynamics Inc., Sunnyvale, CA). The images were analyzed using Progenesis Same Spots (version 4.0, 2010) and Progenesis PG240 (version 2006, Nonlinear Dynamics, Durham, NC) software programs. The computerized analysis included image warping followed by spot finding, background subtraction (average on boundary), matching, and quantification in conjunction with detailed manual checking. The spot % is equal to the spot integrated density above background (volume) expressed as a percentage of the total density above background of all measured spots. A difference is defined as the fold change of spot percentages. The MW and pI values for each spot were determined from algorithms applied to the reference image. The spots of interest were excised and subjected to LC-MS/MS analysis for protein identification at the mass spectrometry facility of Columbia University.

### Sub-cellular Fractionation

Sub-cellular fractionation was performed using the Qproteome Mitochondria Isolation Kit (#37612, Qiagen) according to the manufacturer’s protocol. Briefly, cells were lysed in the provided lysis buffer and then centrifuged at 1,000 x *g* for 10 minutes at 4°C. The supernatant was collected as the cytosolic fraction. The remaining pellet was re-suspended in ice-cold disruption buffer, passed through a 25-gauge blunt-ended needle 20 times (For HEK293 cells, this step was replaced by vortexing) and then centrifuged at 700 x *g* for 10 minutes at 4°C. The pellet was collected as the nuclear fraction. The supernatant was transferred to a new tube and then centrifuged again at 6,000 x *g* for 10 minutes at 4°C to pellet the crude mitochondria. The mitochondrial fraction was further purified using the high-purity preparation protocol described in the manual.

### Immunoprecipitation and Immunoblotting

For the Flag-SIRT1 immunoprecipitation (IP) assays, HEK293 cells were transfected with either pECE empty vector or pECE-Flag-SIRT1 using Lipofectamine-LTX reagent (Invitrogen). Forty-eight hours later, the cells were harvested, and WCLs were prepared and incubated with anti-Flag M2 Affinity Agarose Gel (Sigma) at 4°C in NETN buffer (50 mM Tris-HCl [pH 8.0], 100 mM NaCl, 1 mM EDTA, 0.5% NP40) overnight. For the ATP6V1B2 IP assays, an antibody against endogenous ATP6V1B2 (ab73404, Abcam) was used under the same condition as described above. The immunoprecipitated proteins were then subjected to immunoblotting analysis using the indicated antibodies. For the over-expression of His/Myc-tagged full-length SIRT1, HEK293 cells were transfected with either pcDNA3 empty vector or pcDNA3-SIRT1-His/Myc using Lipofectamine-LTX reagent (Invitrogen). For the western blot analysis, equal amounts of protein samples were resolved by SDS-PAGE at the appropriate percentages, transferred onto PVDF membranes, and probed with antibodies recognizing SIRT1 (#2028, Cell Signaling), Flag tag (F3165, Sigma), His tag (#2365S, Cell Signaling), Acetyl-K382-p53 (#2525, Cell Signaling), p53 (sc-101361, Santa Cruz), ATP6V1B2 (ab73404, Abcam), acetyl-lysine (#9441, Cell Signaling), HK-1 (#2024, Cell Signaling), Histone H2A (#2595, Cell Signaling), β-tubulin (#05–661, Millipore) and Calnexin (sc-11397, Santa Cruz).

### Immunocytochemistry and Immunofluorescence Analysis

3T3-L1 cells were seeded on coated cover slips, allowed to differentiate for 5 days and then fixed with 3.7% paraformaldehyde for 24 hours at 4°C. The fixed cells were then permeabilized with PBS containing 0.6% Triton X-100 for 1 hour at room temperature and subsequently blocked with 1% BSA solution containing 10% calf serum for 1 hour. Immunofluorescence analysis was carried out using a SIRT1 antibody (#2028, Cell Signaling), followed by Alexa Fluor 568-labeled anti-mouse secondary antibody (Invitrogen). Nuclei were stained with DAPI (Molecular Probes). The mounted samples were visualized under a confocal microscope (Nikon).

### *In Vitro* Deacetylation Assay

The lysine deacetylase activity of the full-length SIRT1 (#524743, Calbiochem) was examined using the SIRTainty Class III HDAC Assay kit (Millipore) with substrates including human p53 acetylated peptide (ab37536, Abcam) and ATP6V1B2 (H00000526-P01, Abnova). Recombinant histone H3 (M2503S, NEB) was used as a negative control for lysine acetylation. The levels of lysine deacetylation were determined by measuring the intensities of the fluorescent signals generated by free ammonia, a byproduct of the sirtuin-catalyzed lysine deacetylation reaction, using a fluorescence plate reader (Tecan).

### Cell Toxicity Assay

The cell toxicities of the SIRT1 modulator treatments were examined using the lactic dehydrogenase (LDH)-based *In Vitro* Toxicology Assay Kit (#TOX7, Sigma). Briefly, 3T3-L1 preadipocytes were treated with 10 μM EX527, SRTCX1002, or SRTCZ1001 for 24 hours. Then, the culture media were collected for LDH release measurements using a spectrophotometer as per the manufacturer’s recommendations.

## Results

### Optimization of SIRT1 modulator treatment

SIRT1 plays a key role in the control of multiple metabolic processes through protein deacetylation. To further elucidate the molecular mechanism by which SIRT1 contributes to metabolic regulation, we sought to identify its downstream targets in murine 3T3-L1 adipocytes through a non-biased proteomic approach. In this study, we aimed to modulate SIRT1 deacetylase activity using small molecules, to examine the state of lysine acetylation after treatment and to identify targets with dramatic changes in acetylation.

**Fig 1:**
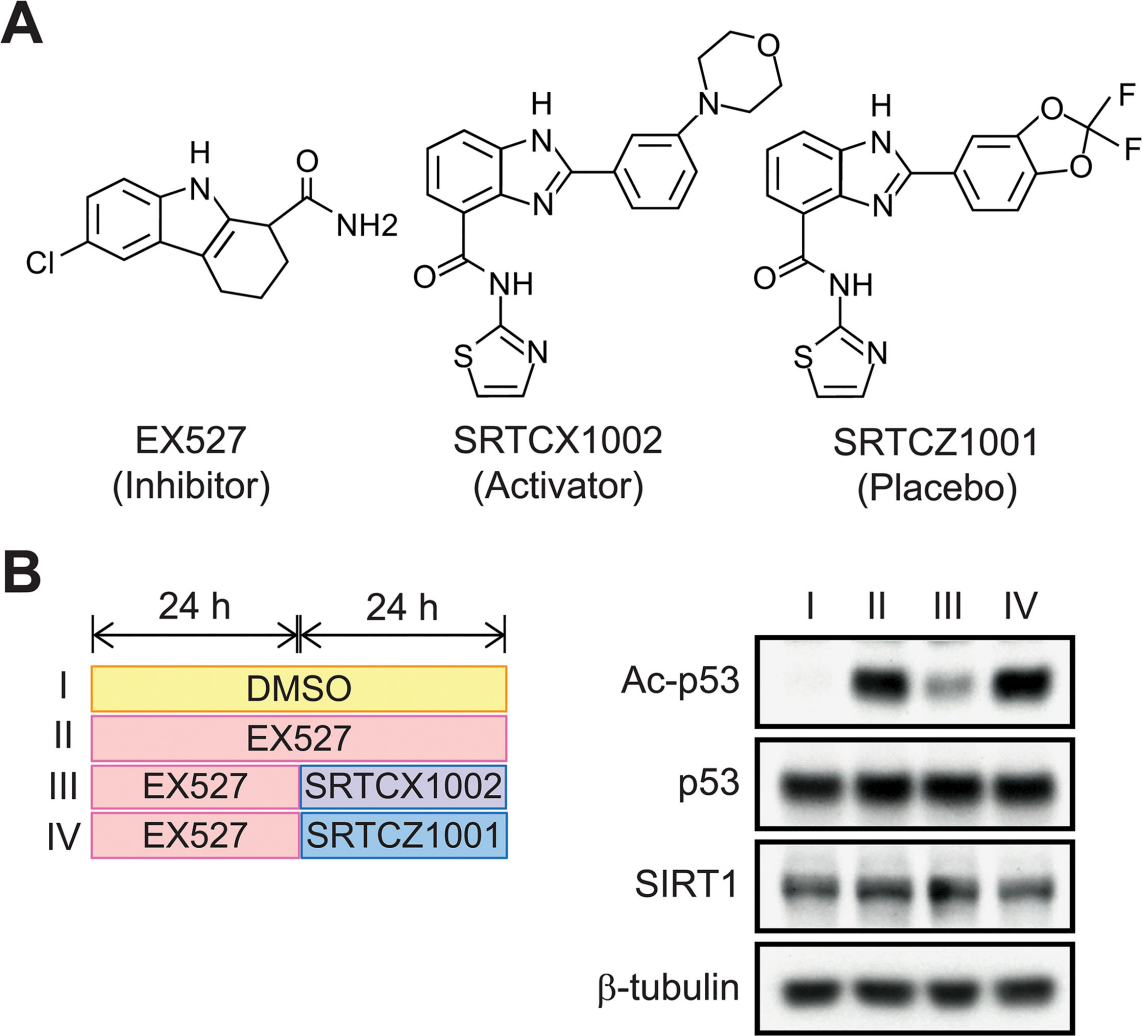
Validation of SIRT1 modulators in HEK293 cells. **(A)** Chemical structures of the SIRT1 modulators EX527 (an inhibitor), SRTCX1002 (an activator) and SRTCZ1001 (a placebo). **(B)** HEK293 cells were treated with DMSO (blank control, I) or EX527 (II) for 48 hours or EX527 for 24 hours and then switched to SRTCX1002 (III) or SRTCZ1001 (IV) treatment for an additional 24 hours (left panel). The acetylation levels of p53 lysine-382, a known target of SIRT1 deacetylation, were examined by western blotting using a specific antibody. The p53 and SIRT1 protein levels were also determined by western blotting. β-tubulin was used as a loading control.

Toward this end, we used specific SIRT1 modulators that were validated and published previously, including EX527 (an inhibitor) [27, 28], SRTCX1002 (an activator) and SRTCZ1001 (a placebo) [29–31] (Fig 1A). To validate the action of these small molecules in our hands, they were used to treat HEK293 cells, as illustrated in Fig 1B (left panel). Then, the levels of p53 lysine-382 acetylation, a known target of SIRT1 deacetylation, were evaluated by western blotting. As expected, EX527 treatment led to a dramatic increase in p53 acetylation, indicating strong inhibition of SIRT1 deacetylase activity (Fig 1B, right panel). Not surprisingly, the level of p53 acetylation was significantly reduced upon SRTCX1002 treatment, demonstrating that SRTCX1002 is a strong SIRT1 activator. In contrast, SRTCZ1001 treatment had no notable effect on the p53 acetylation level. In parallel, we confirmed that neither p53 nor SIRT1 protein levels were altered by SIRT1 modulator treatment (Fig 1B).

After confirming the effects of these SIRT1 modulators on SIRT1 deacetylase activity, we next examined the toxicity of these chemicals in 3T3-L1 cells. In this experiment, actively proliferating 3T3-L1 preadipocytes were treated with 10 μM EX527, SRTCX1002, or SRTCZ1001 for 24 hours and subsequently visualized under a microscope to determine morphological changes or examined for lactic dehydrogenase (LDH) release using an *In Vitro* Toxicology Assay Kit. Compared to the blank control (DMSO treatment), no notable morphological changes in 3T3-L1 cells were observed after SIRT1 modulator treatments (S1A Fig). In addition, LDH release was unaltered by treatment with these chemicals (S1B Fig). We thus concluded that these SIRT1 modulators were highly effective at modulating SIRT1 deacetylase activity without affecting either the SIRT1 protein level or cell viability at the concentration tested.

### Identification of novel SIRT1 targets in 3T3-L1 mature adipocytes

**Fig 2:**
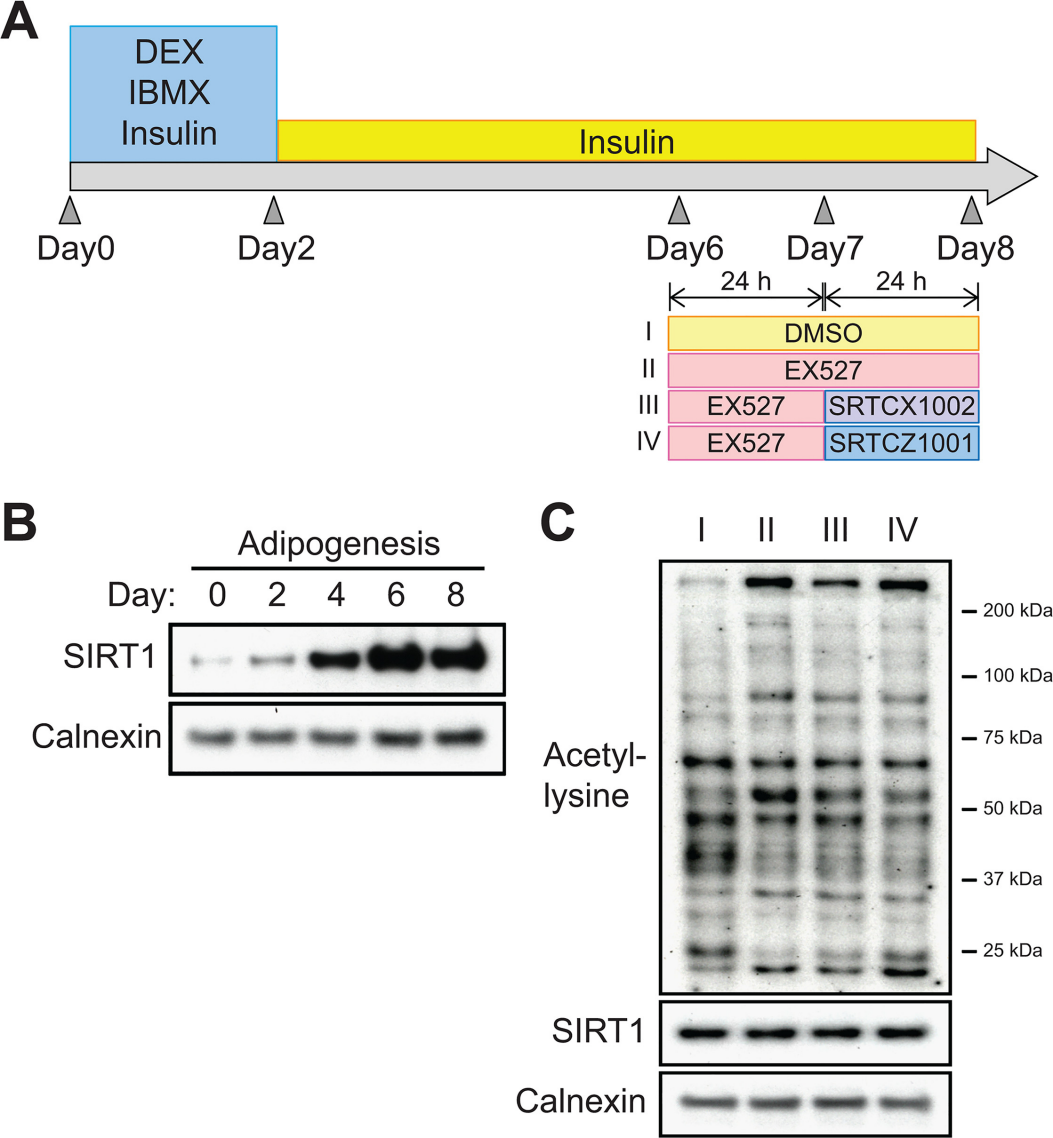
Patterns of global protein acetylation after SIRT1 modulator treatments in 3T3-L1 mature adipocytes. **(A)** Schematic illustration of the experimental procedure. Briefly, 3T3-L1 cells were differentiated into mature adipocytes for 6 days. Then, the mature adipocytes were treated with SIRT1 modulators as follows: (I) DMSO for 48 hours; (II) EX527 for 48 hours; (III) EX527 for 24 hours followed by a switch to SRTCX1002 for 24 hours; and (IV) EX527 for 24 hours followed by a switch to SRTCZ1001 for 24 hours. **(B)** SIRT1 protein levels were examined at the indicated times during 3T3-L1 adipogenesis by western blotting using an antibody against the C-terminal portion of SIRT1. Calnexin was used as a loading control. **(C)** Patterns of global protein acetylation in 3T3-L1 mature adipocytes after SIRT1 modulator treatments, as described in panel (A). The protein acetylation state was evaluated using a pan-acetyl-lysine antibody. The SIRT1 protein level was evaluated using an antibody against the C-terminal portion of SIRT1. Calnexin was used as a loading control.

To obtain mature adipocytes, 3T3-L1 preadipocytes were differentiated *in vitro* for 6–8 days until a significant amount of lipid droplets was observed. Subsequently, the differentiated 3T3-L1 cells were treated with SIRT1 modulators as illustrated in Fig 2A. We chose to begin the treatment on Day 6 because the protein level of SIRT1 peaks at this time point and is maintained at a high level until Day 8 of 3T3-L1 adipogenesis (Fig 2B). Then, the SIRT1 modulator-treated cells were harvested, and the pattern of global protein acetylation was examined by western blotting using a pan-acetyl-lysine antibody. As shown in Fig 2C, an increase in the lysine acetylation of some proteins was observed after EX527 treatment. Again, the SIRT1 protein level was unaffected by SIRT1 modulator treatment in mature adipocytes (Fig 2C).

**Fig 3:**
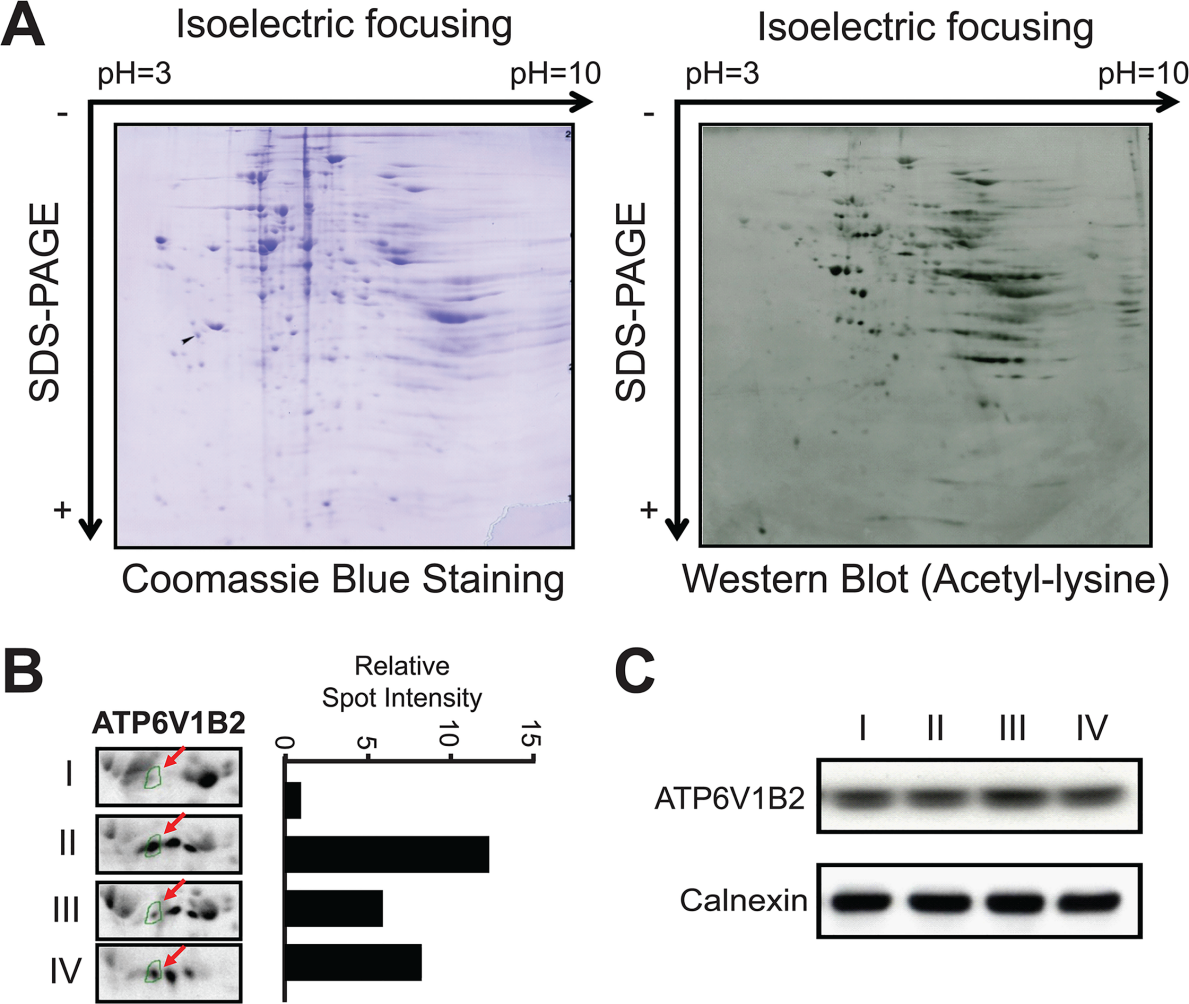
Proteomic analysis of SIRT1 modulator-treated 3T3-L1 mature adipocytes identified ATP6V1B2 as a potential SIRT1 target. **(A)** Representative 2D-PAGE gel transfer blot stained with Coomassie blue and utilized in western blot analysis using a pan-acetyl-lysine antibody. **(B)** Western blot spots corresponding to ATP6V1B2 showed differential acetylation states upon SIRT1 modulator treatments, as described in Fig 2A. The spot intensities were quantified and are shown as a bar graph in the right panel. The value for the blank control was arbitrarily set as one. **(C)** The protein level of ATP6V1B2 was examined by western blot analyses after SIRT1 modulator treatment of 3T3-L1 mature adipocytes. Calnexin was used as a loading control.

Next, we prepared WCLs from the treated 3T3-L1 mature adipocytes (Fig 2A) and separated them by two-dimensional gel electrophoresis (2-DE) (Fig 3A). Subsequently, these protein samples were transferred to a PVDF membrane and probed with a pan-acetyl-lysine antibody. In parallel, identical samples were separated and transferred to PVDF membranes for Coomassie blue staining. We expected that the acetylation level of a potential target of SIRT1 deacetylation would increase after SIRT1 inhibitor EX527 treatment (Treatment II vs. Treatment I) and subsequently decrease if EX527 was substituted with the SIRT1 activator SRTCX1002 (Treatment III vs. Treatment II). However, if EX527 is replaced by the placebo SRTCZ1001, the acetylation level of the candidate protein should not change much (Treatment IV vs. Treatment II) (Fig 2A). Based on this consideration, we first quantified the Spot % of 264 spots using computational analysis (Materials and Methods) and calculated the fold changes in their signal intensities as well as their statistical significance (S1 Table). Subsequently, we selected 7 spots with acetylation level changes that best fit our criteria and located them on the Coomassie blue stained PVDF membrane by matching the membrane to the western blot film. These spots were excised and their identities were then determined by LC-MS/MS analysis. Interestingly, several proteins involved in ATP hydrolysis and FAO were among the candidates for SIRT1 deacetylation (S2 Table). We chose ATP6V1B2 for further validation. As shown in Fig 3B, the acetylation level of ATP6V1B2 increased significantly after SIRT1 inhibitor treatment and subsequently decreased upon SIRT1 activator treatment; however, the acetylation level changed less when the SIRT1 inhibitor was replaced by the placebo. To rule out the possibility that the changes in the acetylation level observed through western blotting were caused by changes in the protein level after the treatments, we examined the protein expression of ATP6V1B2 using its corresponding antibody. We found that the protein level of ATP6V1B2 was unaltered after SIRT1 modulator treatment in 3T3-L1 mature adipocytes (Fig 3C).

### SIRT1 directly interacts with ATP6V1B2

**Fig 4:**
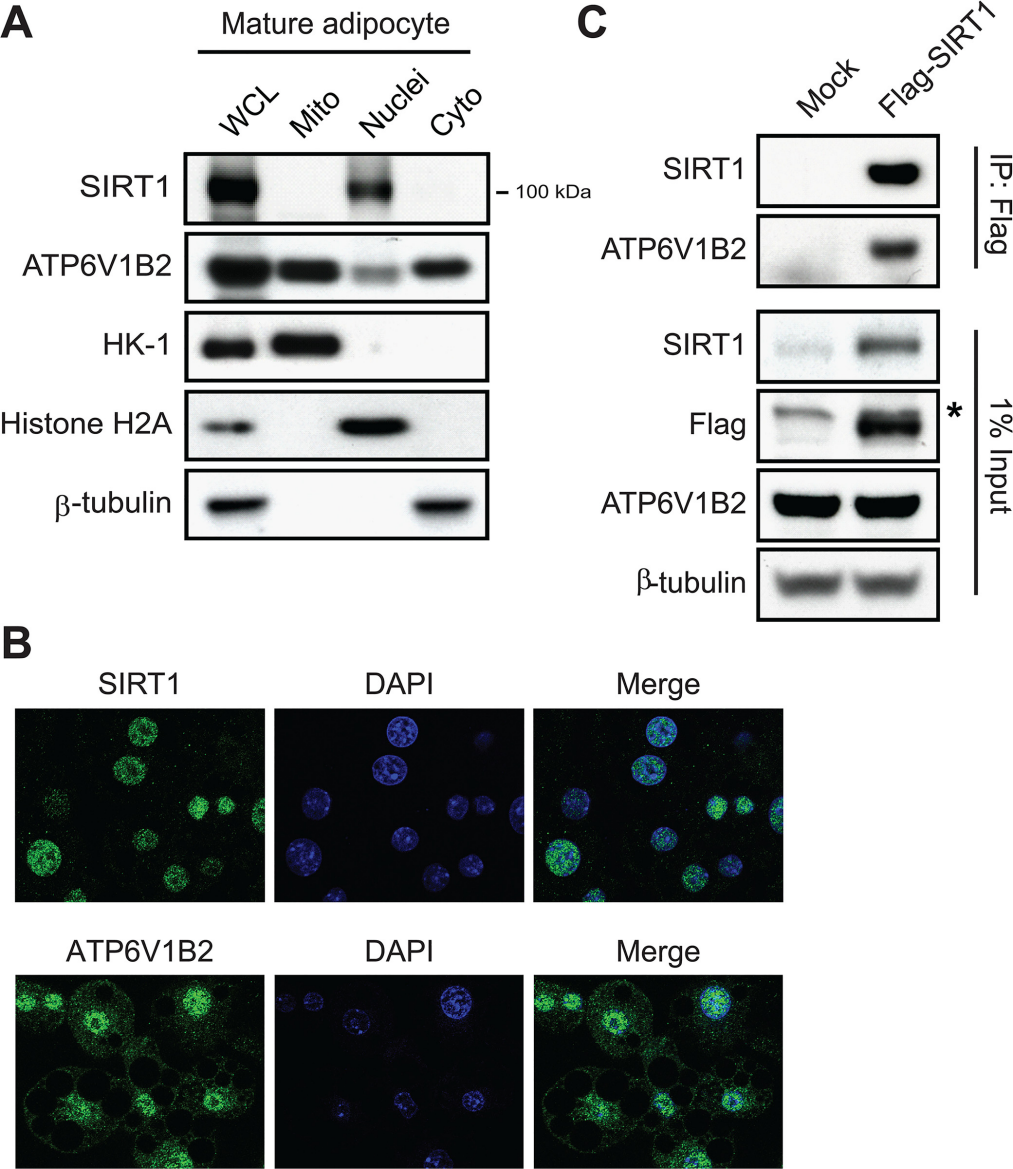
SIRT1 directly interacts with ATP6V1B2. **(A)** The sub-cellular localization of SIRT1 and ATP6V1B2 was determined by western blot analyses of fractionated mitochondrial (Mito), nuclear (Nuclei), and cytosolic (Cyto) samples from 3T3-L1 mature adipocytes. Markers of the mitochondrial (HK-1), nuclear (histone H2A) and cytosolic (β-tubulin) fractions were examined by western blotting to verify the purity of our preparations. Whole-cell lysate (WCL) was included as a positive control. **(B)** Sub-cellular localization of SIRT1 and ATP6V1B2 was examined by immunofluorescence analysis of 3T3-L1 mature adipocytes using a SIRT1 antibody and an ATP6V1B2 antibody, respectively. Nuclei were stained with DAPI. **(C)** Flag-tagged full-length SIRT1 (Flag-SIRT1) was expressed in HEK293 cells and immunoprecipitated by M2-agarose. The interaction between ATP6V1B2 and Flag-SIRT1 was examined by western blot analysis of ATP6V1B2 in the Flag-IP sample. The protein levels of SIRT1, Flag-SIRT1 and ATP6V1B2 were assessed by western blotting using the corresponding antibodies. β-tubulin was used as a loading control. The asterisk indicates a non-specific band detected by the Flag antibody.

After identifying ATP6V1B2 as a potential target for SIRT1 deacetylation, we sought to determine the sub-cellular localization of this protein in 3T3-L1 adipocytes, with the aim of verifying its co-localization with the SIRT1 deacetylase. Toward this end, we fractionated WCLs from 3T3-L1 adipocytes into mitochondrial, nucleic, and cytosolic fractions, and the purity of our preparations was confirmed by examining markers of the mitochondrial (hexokinase-1, HK-1), nuclear (histone H2A) and cytosolic (β-tubulin) fractions using western blotting (Fig 4A). Consistent with previous findings, ATP6V1B2 was found in the mitochondrial and cytosolic fractions (Fig 4A). In addition, it was also found in the nucleus of 3T3-L1 adipocytes. SIRT1 has generally been considered to be a nuclear protein [32]; however, recent studies have shown that it contains two nuclear export signals and that it is found in the cytoplasm [22] and mitochondria [33]. To determine the sub-cellular localization of SIRT1 in 3T3-L1 adipocytes, we used an antibody that specifically recognized its C-terminal region in the western blot analysis because a shorter, C-terminal version of SIRT1 was recently identified in the WAT of mice fed a high-fat diet [34]. As shown in Fig 4A, the C-terminal SIRT1 antibody only detected full-length SIRT1 (~ 100 kDa) in 3T3-L1 adipocytes, and full-length SIRT1 localized exclusively to the nucleus. To further confirm the nuclear localization of SIRT1 in 3T3-L1 adipocytes, we used immunocytochemistry. When the SIRT1 antibody was used in this analysis, the immunofluorescence signal was only detected in the DAPI stained nucleus (Fig 4B). In contrast, ATP6V1B2 antibody detected this protein in both the nucleus and cytosol (Fig 4B). Next, we tested the direct interaction between SIRT1 and its potential target by co-immunoprecipitation (co-IP) experiments. In this assay, a Flag-tagged, full-length SIRT1 protein was overexpressed in HEK293 cells and immunoprecipitated using the Flag antibody. The overexpression of Flag-SIRT1 was confirmed by western blotting using two independent antibodies (Anti-SIRT1 and anti-Flag) (Fig 4C). The overexpression of Flag-SIRT1 had no notable effect on the protein level of ATP6V1B2. In the co-IP experiment, Flag-SIRT1 was successfully pulled down, and ATP6V1B2 was clearly detectable in the IP sample, indicating a direct interaction between SIRT1 and ATP6V1B2 in the nucleus (Fig 4A and 4C).

### SIRT1 deacetylates ATP6V1B2

**Fig 5:**
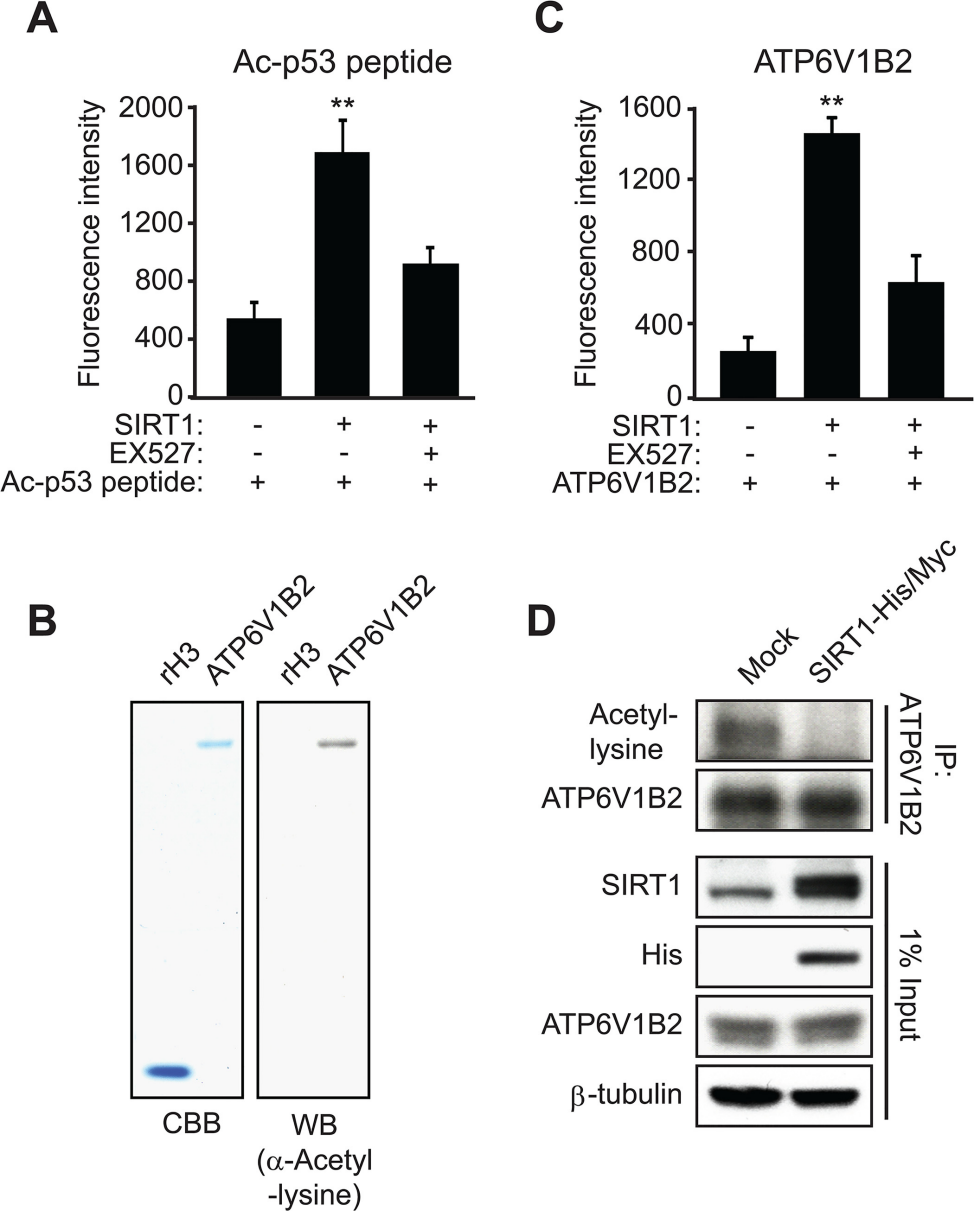
SIRT1 deacetylates ATP6V1B2 *in vitro* and *in vivo*. **(A)** Acetylated p53 (Ac-p53) peptide was deacetylated by SIRT1 *in vitro*. The deacetylation level of the peptide was evaluated by measuring the fluorescent signal from free ammonia, a byproduct of the deacetylation reaction catalyzed by sirtuin enzymes. EX527, a specific SIRT1 inhibitor, significantly reduced the ability of SIRT1 to deacetylate the Ac-p53 peptide. **(B)** ATP6V1B2 protein purified from wheat germ is acetylated at lysine residues. The acetylation status of ATP6V1B2 was examined using a pan-acetyl-lysine antibody in the western blot analysis. Recombinant histone H3 (rH3) was used as a negative control for lysine acetylation. The protein levels were determined by Coomassie Brilliant Blue (CBB) staining. **(C)** ATP6V1B2 was deacetylated by SIRT1 *in vitro*. The data presented in this Fig are based on three independent experiments (mean ± SED) (** *P* < 0.01). **(D)** Endogenous ATP6V1B2 was immunoprecipitated by a specific antibody in HEK293 cells with or without over-expression of His/Myc-tagged full-length SIRT1 (SIRT1-His/Myc). The state of lysine acetylation of ATP6V1B2 was examined by western blot analysis using a pan-acetyl-lysine antibody in the IP sample. The protein levels of SIRT1, SIRT1-His/Myc and ATP6V1B2 were assessed by western blotting using the corresponding antibodies. β-tubulin was used as a loading control.

Given that the interaction between SIRT1 and ATP6V1B2 was confirmed by the co-IP experiment, we next questioned whether ATP6V1B2 could be deacetylated by SIRT1 *in vitro*. As a positive control, we first performed the *in vitro* deacetylation assay using a p53 peptide acetylated at lysine-382 as the substrate. Not surprisingly, SIRT1 strongly deacetylated the acetylated p53 peptide, and this activity was inhibited by the SIRT1 inhibitor EX527 (Fig 5A). For the ATP6V1B2 deacetylation assay, we used ATP6V1B2 protein that was purified from wheat germ and confirmed to be acetylated by western blotting using a pan-acetyl-lysine antibody (Fig 5B). In the *in vitro* deacetylation assay, SIRT1 demonstrated strong deacetylase activity toward ATP6V1B2, and this activity was significantly reduced by EX527 treatment (Fig 5C). Moreover, when SIRT1-His/Myc was over-expressed in the HEK293 cells, the level of lysine acetylation of immunoprecipitated ATP6V1B2 was significantly reduced (Fig 5D). But the over-expression of SIRT1 or the change of lysine acetylation state does not affect the nuclear localization of ATP6V1B2 (S2 Fig). In summary, we conclude that SIRT1 directly interacts with and deacetylates ATP6V1B2. Subsequently, we examined the acetylation state of ATP6V1B2 during 3T3-L1 adipogenesis and we found that ATP6V1B2 showed a slight decrease in its acetylation level in mature adipocytes as compared with preadipocytes (S3 Fig).

## Discussion

SIRT1 is involved in the regulation of multiple metabolic pathways by modulating the activities of various transcription factors and metabolic enzymes through lysine deacetylation. In this study, we provided evidence showing that the B subunit of the vacuolar (H^+^)-ATPase, ATP6V1B2, is a SIRT1 target in mouse 3T3-L1 adipocytes. The vacuolar (H^+^)-ATPases play an essential role in controlling the acidification of intracellular compartments such as endosomes, lysosomes and Golgi-derived vesicles in eukaryotic cells [35, 36]. These enzymes are multi-subunit complexes composed of a peripheral V_1_ domain and an integral V_0_ domain. The V_1_ domain is a 640-kDa complex containing eight different subunits and it catalyzes ATP hydrolysis [36]. And the ATP hydrolysis activity of the enzyme is carried out at the catalytic nucleotide binding sites located primarily on the A subunits. Although the B subunits also contain nucleotide binding sites, but these sites are thought to be non-catalytic. In our sub-cellular localization assay, ATP6V1B2 was not only found in the mitochondrial and cytosolic fractions but also found in the nuclear fraction. Apparently this protein interacts with SIRT1 directly inside the nucleus as SIRT1 was only found in the nuclear fraction of the mature adipocytes. Meanwhile, ATP6V1B2 could also be target of lysine deacetylation by mitochondrial and cytosolic histone deacetylases such as SIRT3 and SIRT2. Currently the role of lysine acetylation of ATP6V1B2 is unclear and the biological significance of SIRT1 deacetylation of ATP6V1B2 also awaits further investigation. Nevertheless our observation suggested a potential role of SIRT1 in regulating the function of vacuolar (H^+^)-ATPases in the acidification of intracellular compartments.

In addition to ATP6V1B2, we surprisingly identified several mitochondrial proteins as potential SIRT1 targets in our proteomic analysis. Among them, there are two key enzymes (ECH1 and HADH2) involved in FAO. Given that SIRT1 was not found in the mitochondria in our sub-cellular localization analysis (Fig 4), we reasoned that the acetylation status of these mitochondrial proteins were affected by indirect mechanisms through modulation of SIRT1 deacetylase activity. A recent quantitative acetylome study showed that a number of lysine acetyltransferases are targets of SIRT1 deacetylation and their activities of acetyl group transferring were profoundly affected by SIRT1 mediated deacetylation [20]. So, it's formally possible that the acetylation status of the mitochondrial proteins identified in this study was affected by the altered acetyltransferases activities, or, through an as-yet-unknown mechanism.

## Supporting Information

S1 Fig Treatment with SIRT1 modulators does not affect cell viability in 3T3-L1 preadipocytes. **(A)** Representative microscopic images of 3T3-L1 preadipocytes after 24-hour treatment with DMSO (blank control), 10 μM EX527, 10 μM SRTCX1002, or 10 μM SRTCZ1001. **(B)** Cell viability assays were performed by measuring LDH release from 3T3-L1 preadipocytes using the same drug treatments as described in panel (A). These results are the averages of three independent experiments with standard deviations as indicated by the error bars.
(EPS)

S2 Fig SIRT1 over-expression does not affect the nuclear localization of ATP6V1B2. The nuclear localization of ATP6V1B2 was determined by western blot analysis of purified nuclear fraction from HEK293 cells with or without over-expression of SIRT1-His/Myc. Markers of the nuclear (histone H2A), mitochondrial (HK-1), and cytosolic (β-tubulin) fractions were examined by western blotting to verify the purity of our preparations. Whole-cell lysate (WCL) was included as a positive control.
(TIF)

S3 Fig The acetylation level of ATP6V1B2 decreases slightly during 3T3-L1 adipogenesis. Endogenous ATP6V1B2 was immunoprecipitated from 3T3-L1 preadipocytes (Day 0) and mature adipocytes (Day 8). The acetylation level of ATP6V1B2 was examined by western blot analysis using a pan-acetyl-lysine antibody in the IP sample. The protein level of ATP6V1B2 was assessed by western blotting using the corresponding antibody. Calnexin was used as a loading control.
(TIF)

S1 Table List of the protein spots that showed differential acetylation states upon treatment with SIRT1 modulators. (XLSX)

S2 Table List of the proteins identified by LC-MS/MS that showed differential acetylation states upon treatment with SIRT1 modulators. (XLSX)
