# Supplementary figures and images for: SIRT1 Interacts with and Deacetylates ATP6V1B2 in Mature Adipocytes
Source: PLoS One. 2015 Jul 15;10(7):e0133448. doi: 10.1371/journal.pone.0133448 (PMC4503461; doi:10.1371/journal.pone.0133448)

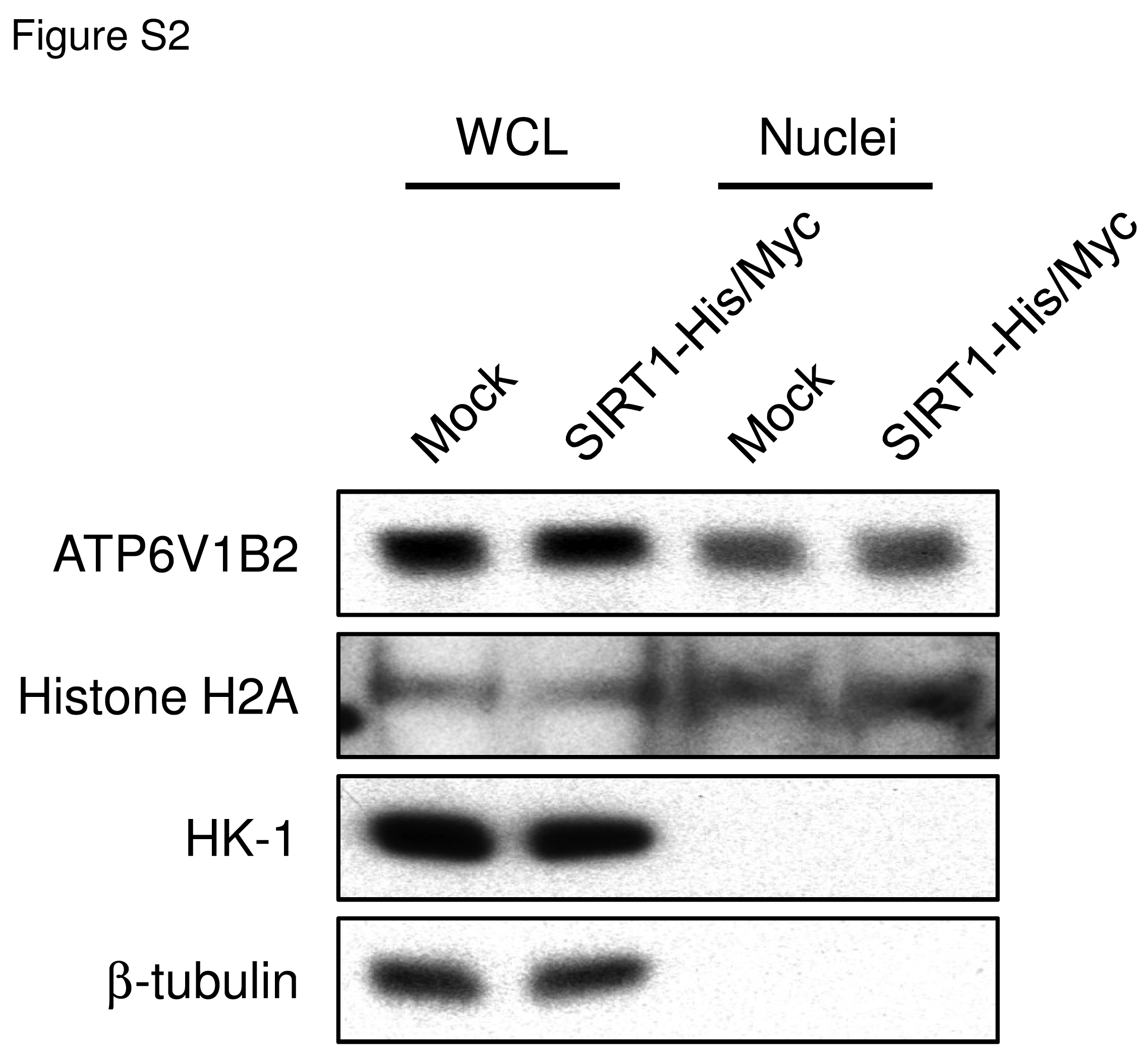

Supplement: S2 Fig — The nuclear localization of ATP6V1B2 was determined by western blot analysis of purified nuclear fraction from HEK293 cells with or without over-expression of SIRT1-His/Myc. Markers of the nuclear (histone H2A), mitochondrial (HK-1), and cytosolic (β-tubulin) fractions were examined by western blotting to verify the purity of our preparations. Whole-cell lysate (WCL) was included as a positive control. (TIF) [file pone.0133448.s002.tif]

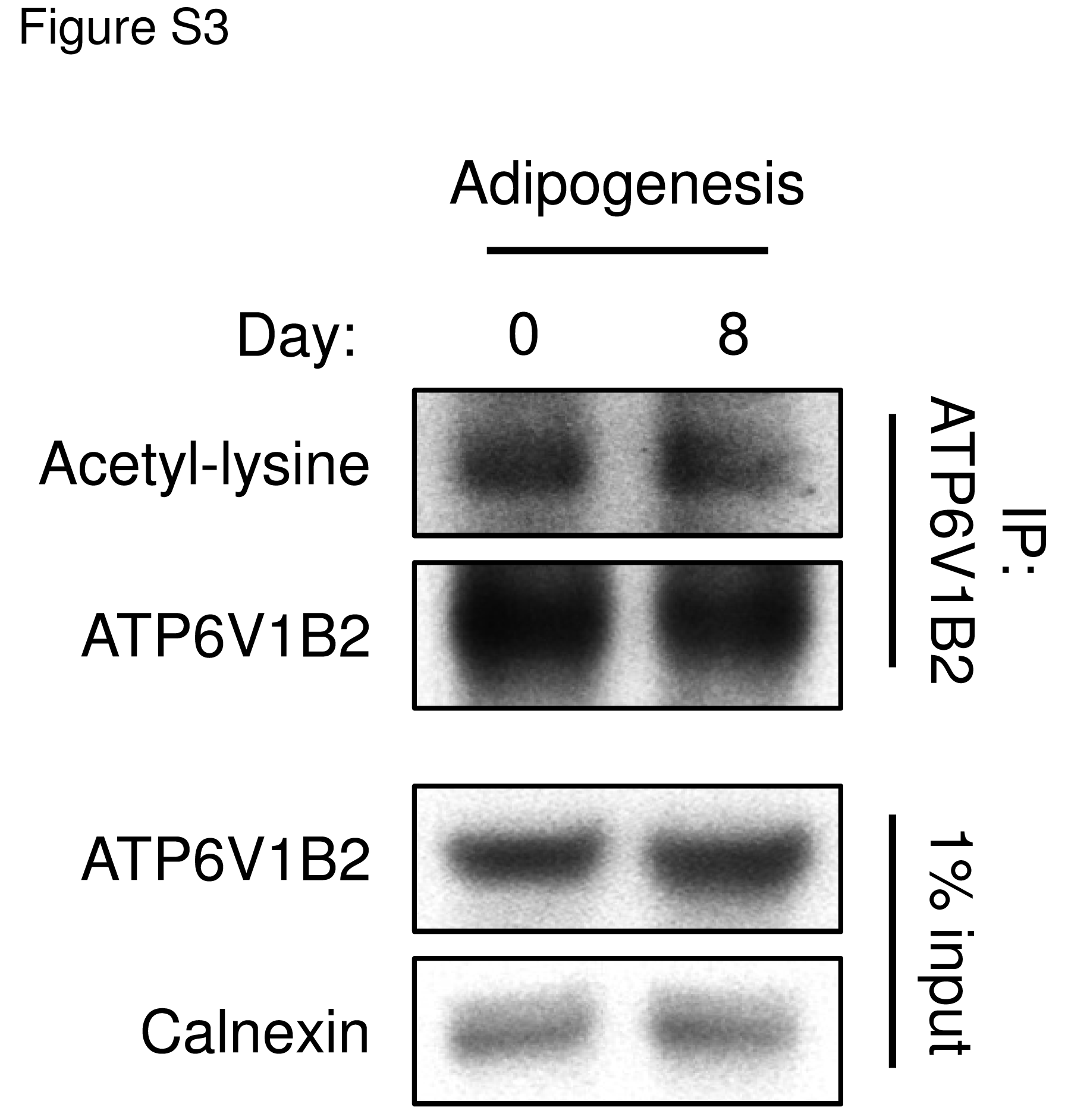

Supplement: S3 Fig — Endogenous ATP6V1B2 was immunoprecipitated from 3T3-L1 preadipocytes (Day 0) and mature adipocytes (Day 8). The acetylation level of ATP6V1B2 was examined by western blot analysis using a pan-acetyl-lysine antibody in the IP sample. The protein level of ATP6V1B2 was assessed by western blotting using the corresponding antibody. Calnexin was used as a loading control. (TIF) [file pone.0133448.s003.tif]
